# A Diverse Virome of Leafroll-Infected Grapevine Unveiled by dsRNA Sequencing

**DOI:** 10.3390/v12101142

**Published:** 2020-10-08

**Authors:** Mamadou L. Fall, Dong Xu, Pierre Lemoyne, Issam E. Ben Moussa, Carole Beaulieu, Odile Carisse

**Affiliations:** 1 Saint-Jean-sur-Richelieu Research and Development Centre, Agriculture and Agri-Food Canada, St-Jean-sur-Richelieu, Quebec, QC J3B 3E6, Canada; dong.xu@canada.ca (D.X.); pierre.lemoyne@canada.ca (P.L.); Issam.Eddine.Ben.Moussa@USherbrooke.ca (I.E.B.M.); odile.carisse@canada.ca (O.C.); 2 Département de Biologie, Université de Sherbrooke, Sherbrooke, QC J1K 2R1, Canada; Carole.Beaulieu@USherbrooke.ca

**Keywords:** grapevine leafroll disease, viromics, dsRNA extraction, virus epidemiology, virus co-occurrence, *Vitis vinifera*, interspecific hybrid

## Abstract

Quebec is the third-largest wine grape producing province in Canada, and the industry is constantly expanding. Traditionally, 90% of the grapevine cultivars grown in Quebec were winter hardy and largely dominated by interspecific hybrid *Vitis* sp. cultivars. Over the years, the winter protection techniques adopted by growers and climate changes have offered an opportunity to establish *V. vinifera L*. cultivars (e.g., Pinot noir). We characterized the virome of leafroll-infected interspecific hybrid cultivar and compared it to the virome of *V. vinifera* cultivar to support and facilitate the transition of the industry. A dsRNA sequencing method was used to sequence symptomatic and asymptomatic grapevine leaves of different cultivars. The results suggested a complex virome in terms of composition, abundance, richness, and phylogenetic diversity. Three viruses, grapevine Rupestris stem pitting-associated virus, grapevine leafroll-associated virus (GLRaV) 3 and 2 and hop stunt viroid (HSVd) largely dominated the virome. However, their presence and abundance varied among grapevine cultivars. The symptomless grapevine cultivar Vidal was frequently infected by multiple virus and viroid species and different strains of the same virus, including GLRaV-3 and 2. Our data show that viruses and viroids associated with the highest number of grapevines expressing symptoms included HSVd, GLRaV-3 and GLRaV-2, in gradient order. However, co-occurrence analysis revealed that the presence of GLRaV species was randomly associated with the development of virus-like symptoms. These findings and their implications for grapevine leafroll disease management are discussed.

## 1. Introduction

The Canadian grape industry is constantly expanding, with a production area of more than 12,000 ha in 2019 for a total revenue of CAD 9 billion dollars[1]. Quebec is the third-largest wine grape producer in Canada in terms of acreage, tonnage and wine grape sales. Quebec’s industry is also increasing the area dedicated to grapevine production. Traditionally 90% of the grapevine cultivars cultivated in Quebec were rustic (winter hardy), with nearly 50% of the grapevine production based on an interspecific hybrid *Vitis* sp. cultivar named Vidal, which was developed in the 1930s, because these hybrid cultivars were able to survive harsh winter conditions and produced mature berries in the short and warm growing season. Over the years, knowledge about the most promising cultivars and constant improvement of techniques favoring the survival of the plants has led to a profound change of the industry. Furthermore, growers’ experience and climate changes offered an opportunity to establish *V. vinifera L.* cultivars, expanding the diversity of wine offered as much for taste and flavor as for the joy of the consumers. However, this increased assortment of grapevines implied various new challenges, including different susceptibility to known diseases and potentially new ones, particularly viruses.

More than 80 viruses and viroids have been discovered over the years to affect grapevine, either singly or as mixed infections [2,3]. Among perennial crops, worldwide, grapevine is considered to host the highest number of viruses [3]. In north-eastern North America, tomato ring spot virus (ToRSV), arabis mosaic virus (ArMV), grapevine leafroll-associated virus (GLRaV), the rugose wood-associated viruses, comprising grapevine Rupestris stem pitting-associated virus (GRSPaV), grapevine virus A (GVA), grapevine virus B (GVB) and grapevine virus D (GVD), grapevine fleck virus (GFkV), grapevine Red Globe virus (GRGV), grapevine fanleaf virus (GFLV) and grapevine red blotch-associated virus (GRBV) are the prominent viruses scrutinized [2]. Among these viruses, grapevine leafroll disease complex (GLD), which is associated with six known viruses, is one of the most prevalent and draws particular attention because of its adverse economic impacts on the grapevine yield [4,5,6,7].

It is known that the symptoms of GLD vary with seasons, cultivars and weather conditions [8,9]. Some infected cultivars can present strong, little or no symptoms, which are well-described in *Vitis vinifera* cultivars and include interveinal reddening, backward rolling of leaf margins and mild chlorosis [8,10,11]. Indeed, some *Vitis vinifera* cultivars, such as Pinot noir, are known to display strong GLD symptoms, whereas the information on symptom expression on hybrid cultivars such as Vidal is scarce and few studies had focused on hybrid cultivars of grapevine [12,13]. This situation is probably due to the fact that GLRs are latent in hybrid grapevines despite the significant impact on fruit production [14].

In Canada, there are few published studies addressing the presence and incidence of grapevine viruses. In 1996, MacKenzie et al. [15] surveyed grapevine viruses in four regions (British Columbia (BC), Ontario (ON), Québec (QC) and Nova Scotia (NS)) across Canada and found ArMV, GFL, GLR-1 and GLR-3 in BC and ON vineyards, whereas only ArMV and GLR-3 were found in QC and GLR-1 and GLR-3 in NS. Incidences of infestation range from 0.06% to 12.2%. Recently, Poojari et al. conducted studies on the incidence of GLR and GRBV in BC [16,17] and surveyed for GLR, GFL, GRBV, *Grapevine pinot gris virus* (GPG) in NS vineyards in *V. vinifera* and *Vitis* interspecific hybrids [13]. In addition, GLR-3 and GRBV were observed in ON vineyards by Xiao et al. [18] in a study aiming at improving methods to detect viruses for use in diagnostics. However, such studies have not been done in QC since 1996 [15], although concerns were raised by viticulturists as the industry expanded by more than 300% from 2003 to 2018, i.e., from 225 ha to 718 ha [19,20]. In fact, suspicious plants showing characteristic symptoms of virus infection have been seen regularly lately, and diagnostic tests revealed the presence of GLR-3, GRBV, GFkV and ToRSV (Fall, unpublished.).

Throughout the scientific literature, there is limited study on the diversity of the virome of grapevine interspecific hybrid cultivars in comparison with the virome of *Vitis vinifera* cultivars [9,13,21]. It is known that some *Vitis vinifera* cultivars, such as Pinot noir, infected with grapevine leafroll viruses had a complex virome and strongly expressed symptoms of GLD [9]. Quebec grape production initiated a transition from the historically-dominant grapevine interspecific hybrid cultivars (e.g., Vidal) to more *Vitis vinifera* cultivars such as Pinot noir. In fact, this cultivar (Pinot noir) saw an 153.2% increase in terms of total hectares cultivated between 2012 and 2017 (Conseil des vins du Québec). This situation raises concerns and research questions. For example, how will the leafroll-infected asymptomatic grapevine hybrid cultivars (e.g., Vidal) that dominated QC grapevine vineyards act as a reservoir and impact the diversity of the virome of the *Vitis vinifera* grapevine cultivars (e.g., Pinot noir)? As *Vitis vinifera* cultivars are more prone to express symptoms of GLD, the QC vineyard transition from hybrid cultivars to *V. vinifera* cultivars should be approached with caution. There are no methods to efficiently control viruses in vineyards. Chemical control against vectors is costly and might not adequately regulate these populations as well as raising environmental concerns about their usage. Most of our actions in agriculture to cure diseases are in fact done in a reactive fashion, i.e., actions are taken to control the disease once it is established on the crop. In the case of the relationship between grapevines and viruses, it is essential to switch from pathosystems to a more holistic view of the system where grapevine and its virome constitute a micro-ecosystem. From this standpoint, we would benefit from increasing our knowledge on disease epidemiology, anticipating potential threats and designing knowledge-based mitigation strategies before virus epidemics become endemic and difficult to manage [22,23]. Therefore, the objectives of the present work were to characterize the virome of the interspecific hybrid cultivar and compare it to the virome of *V. vinifera* cultivar. Specifically, the aims were to (i) characterize the diversity of the virome of leafroll-infected leaves of different grapevine cultivars under north-eastern weather conditions, (ii) investigate the association between viruses and symptom development and (iii) determine the genetic diversity of viruses and viroids that were detected for the first time in QC.

Here, we report the complexity and the diversity of the virome of grapevine leafroll-infected cold-hardy vines cultivars (e.g., interspecific hybrids Vidal) in comparison with the cold- and virus-sensitive cultivar *Vitis vinifera* (Pinot noir) [4,24,25]. In the hybrid cultivar Vidal, GRSPaV dominated the virome with 61% of viral population, whereas GLRaV-3 and GLRaV-2 represented 13% and 18% of the viral population, respectively. In the cultivar Pinot noir, GLRaV-2 dominated the virome with 43% of the viral population, whereas GRSPaV, GLRaV-3 and hop stunt viroid (HSVd) represented 7%, 38% and 8% of the viral population, respectively.

## 2. Materials and Methods

All statistical tests and graphics were performed in R software, version 3.6.3. The following packages were used: Precrec, ggplot2, eesim, picante, ape, vegan, ade4, permute, lattice, nlme, Complexheatmap (Bionconductor), Cluster, Heatplus, gplots, RcolorBrewer, circlize, colorspace, Getoplong, dendextend (https://cran.r-project.org/).

### 2.1. Plant Material

**Figure 1:**
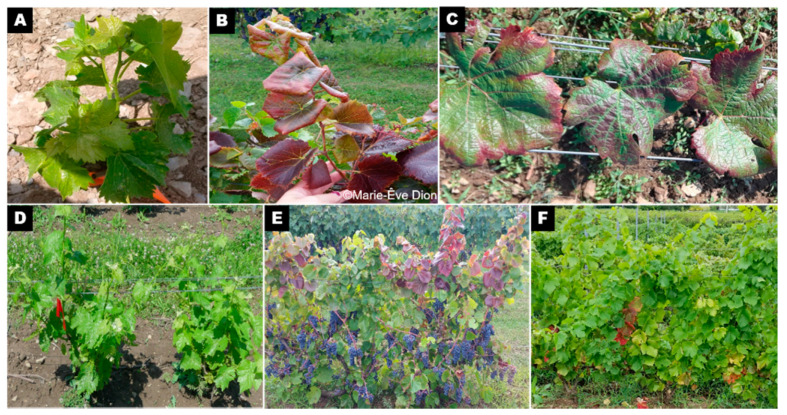
Asymptomatic and symptomatic leaves observed in different cultivars that were positive for grapevine leafroll-associated virus (GLRaV) (GLRaV-2 or 3 or both) in Quebec, Canada. Cultivar Vidal positive to GLRaV without symptom expression (**A**,**D**), cultivar DM85 positive to GLRaV with leafroll symptom expression (**B**,**E**) and cultivar Pinot noir positive to GLRaV with leafroll symptom expression (**C**,**F**).

Leaf samples of asymptomatic and symptomatic leafroll-infected grapevine were collected from three different vineyards in Quebec, Canada (Figure 1). The presence of GLR was confirmed by RT-PCR using primers described by Xiao et al. [26] and Poojari et al. [27]. The first vineyard was at the Agriculture and Agri-Food Canada’s experimental farm in Frelighsburg (Fr, latitude 45°03′12″ N; longitude 72°51′42″ W), the second at a commercial vineyard in Hemmingford (Hem, latitude 45°02′50.99″ N; longitude 73°35′4.04″ W), and the third at a commercial vineyard in Saint-Jacques-le-Mineur (SJM, latitude 45°16′39″ N; longitude 73°25′4.82″ W). The frequencies of sampled grapevine cultivars were 50%, 36%, 7%, 4% and 3% of Vidal (hybrid), Pinot noir (*Vitis vinifera*), Marechal Foch (hybrid), DM85 (hybrid), Seyval blanc (hybrid), respectively. A total of 140 composite samples from 28 grapevine plants (five entire leaves with petiole per plant) were collected per plant between July and September 2018 and 2019 and placed in a plastic bag or a sterile 50-mL centrifuge tube and brought back to the laboratory for cold storage at −20 °C. Leaves were washed with distilled water and roughly crushed before being homogenized in a liquid-nitrogen-cooled 50-mL stainless-steel (SS) grinding jar with one 20–25-mm SS ball with a Retsch tissue lyser MM 400 (Retsch, Haan, Germany). The powdered leaves (2.5–3 g), were then transferred in sterile 50-mL centrifuge tubes and kept at −80 °C until proceeding with double-stranded RNA (dsRNA) extraction.

### 2.2. dsRNA Extraction, Libraries Construction and Sequencing

Extraction of dsRNA from grapevine leaves was performed based on a modified version of the technique from Kesanakurti et al. [28]. Differences brought to the protocol were: black bean (*Phaseolus vulgaris*, Black Turtle Baking Beans (Vesey, York, PEI, Canada) was added as positive control to the homogenized sample-extraction buffer mix at a final concentration of 1% (*w*/*w*), and DNase I and RNase T1 digestions were made after the final dsRNA elution. The cultivar of *P. vulgaris* contains distinct endornavirus species, including *Phaseolus vulgaris endorvirus1* (PvEV1), which was used to assess the efficiency of dsRNA extraction and for monitoring of correctness of high throughput sequencing results. Then digestion was stopped with EDTA 50 mM for 10 min at 65 °C. The dsRNA was denatured at 99 °C for 5 min in the presence of 1 µL of 60 µM random primers and 1 µL of 10 mM dNTP and 4 µL First Strand buffer, and then reverse transcribed with 400 U of Superscript III (Invitrogen) in a final volume of 20 µL for 50 min at 65 °C. The second-strand DNA was synthetized using Klenow polymerase DNA (Promega, Canada) treated with RNase H to get rid of any remaining RNA hybrids, and then cleaned using Agencourt AMPure XP magnetic beads (Beckman-Coulter). Libraries (28, 300 bp mean insert size) were constructed using the Nextera XT DNA library prep kit (Illumina) with 1 ng of input double-stranded cDNA. MiSeq 2 × 250 cycle paired-end sequencing was conducted using MiSeq Reagent Nano Kits v2 in the Illumina Miseq sequencer. The black bean added to all grapevine samples was sequenced alone to differentiate its virome from the grapevine virome associated with each sample.

### 2.3. Bioinformatic and Statistical Analysis

#### 2.3.1. Raw Data Treatment

The raw data fastq files were demultiplexed and checked for sequencing quality using fastQc (https://www.bioinformatics.babraham.ac.uk/projects/fastqc/). Removal of adapter sequencing and reads quality trimming (minimum quality score of 20) were performed using Trimmomatic V.0.32 [29]. Paired fastq files were imported in two different pipelines developed to detect and discover viruses. The first pipeline used was Virtool, an in-house implemented pipeline developed by Rott et al. [30], and the second pipeline was VirFind, an online tool developed by Ho and Tzanetakis [31]. From Virtool, the depth, coverage and weight were deducted. The depth represents the number of times a genome is covered by mapped reads, the coverage measures the percentage of the mapped reads that cover the viral genome, and the weight represents the proportion of reads mapping to a virus that is proportional to the titer. Consensus virus detected by both pipelines was considered as a positive detection. In general, a coverage greater than 0.5 and a weight greater than 0.001 were considered as positive detection of a given virus. In addition, only samples with positive detection of the positive control virus (PvEV1) were conserved for further analysis. The mean proportion of viral read that mapped for a given virus (MPVR), total number of symptomatic leaves (TNSL) associated with a given virus, mean depth (MD), mean depth relative to the depth of the positive control virus (MDRC), mean relative abundance (MRA) and mean weight (MW) were calculated for each virus. The depth was used as a measure of absolute abundance and, since many multivariate methods are sensitive to the total abundance [32], the relative abundance of each detected virus in each sample was calculated using a function of the vegan package. Vegan functions (specpool, estimateR) were used to estimate virus species richness based on incidence in sample plants. The richness Z score and the richness of each detected virus relative to the richness of the positive control virus (PvEV1) were calculated.

#### 2.3.2. Analysis of the Diversity of the Virome and Association between Viruses and Symptom Development

Because grapevine is subject to infection by more than 80 different viruses and viroids, obtaining the ideal number of samples to capture the diversity is challenging. Therefore, the virus species accumulation and abundance as function of virus species rank curves were made using the vegan and ade4 packages to assess the robustness of our sampling effort.

After standardization of the data using the generic function “scale” (R built-in command) to make variables comparable, the distance matrix was calculated, and functions of the ComplexHeatmap package with clustering were used to visualize the heatmap made from Pearson correlation analysis showing the association between variables (MPVR, MW, TNSL, MDRC, MD, MRA and genome size [GS]) and virus species. The pam function of the cluster package was used for partitioning of the data into k clusters around Medoids (k-Medoids, (k = 3)) because it is less sensitive to outliers compared to k-means [33]. In addition, the matrix of data displaying the normalized relative abundance with virus species in columns and the grapevine samples in rows was used to generate a heatmap and a hierarchical clustering to classify virus species and grapevine samples into different groups. The Bray–Curtis dissimilarity matrix and average linkage distance were generated using the vegan package.

To investigate the degree to which viruses species, leafroll symptoms and grapevine cultivars are negatively or positively associated with each other, a recently-published co-occurrence model that is metric-free, distribution-free and randomization-free was used [34,35]. The model determined the probability that observed frequency of co-occurrence between two events (e.g., virus or symptoms presence in a given sample) is significantly greater (large, Pgt) than expected (meaning a positive association), significantly less (small, Plt) than expected (meaning negative association), or not significantly different and approximately equal to expectation (meaning random association) [34,35]. Therefore, Pgt < 0.05 is considered to highlight a positive co-occurrence between the two events, and Plt < 0.05 is interpreted as a negative co-occurrence between the two events. The R package co-occur was used to determine the co-occurrence, and the probability (*P*) that two species (viruses or grapevine species) co-occur at j number of sampling units (a grapevine plant) is calculated as follows:

$$P_{j}=\frac{\left(N_{\begin{array}{c} 1\\ j \end{array}}\right)X\;\left(\begin{array}{cc} N- & N_{1}\\ N_{2}- & j \end{array}\right)\;}{\left(\begin{array}{c} N\\ N_{2} \end{array}\right)}$$

where *N*_1_ is the number of sampling units where the first event occurs, *N*_2_ is number of sampling units where the second event occurs, and *N* is the total number of sampling units. To gain more robustness, additional data were gathered from five published manuscripts using the same NGS methodologies to characterize the virome of grapevine plants [9,36,37,38,39]. In summary, a total of 66 sampling units and 1452 points of comparison were obtained and used in this analysis. Additionally, any event pairs that were expected to share less than one sampling unit were removed from the analysis.

To determine the genetic diversity of important viruses and viroids that were detected for the first time in QC, phylogenetic trees were generated from nucleotide alignments of partial (concatenated, details in Tables S2 and S3, respectively) or complete sequences of the genome from samples that were positive for a given virus. Maximum likelihood algorithms were carried out using bootstrap (1000 pseudo-random iterations) to assess the confidence of the branching pattern, and the phylogram package in R was used. Full genomes from different clades and geographical regions were downloaded from GenBank and added to the tree. Selection of reference genomes was performed by running a BLAST in GenBank using an isolate as query and from the result page; the genome with the highest alignment scores was chosen. One genome of the same cluster with selected isolate and at least two other genomes from different clusters were selected. The Newick format file was exported to the interactive tree of life platform for better visualization and creation of high-quality figures [40]. To display an overview of the diversity among the sequences, pairwise nucleotides comparison analysis was used to generate percentage of identity and distance for each pair of sequences using CLC Main Workbench software (V 20.0.3). The percentage of identity was calculated by dividing the number of identical residues in the alignment positions with the total number of overlapping alignment positions between the two sequences. The distance was measured using the Jukes–Cantor distance between the two sequences and is the proportion between identical and overlapping alignment positions between two sequences.

## 3. Results

### 3.1. Diversity of Virome in Leafroll-Infected Leaves of Different Grapevine Cultivars

The grapevine plants infected with leafroll expressed different intensity of symptoms. The cultivar Vidal, an interspecific hybrid, expressed little to no visible leafroll symptoms in comparison with other cultivars of *Vitis vinifera* (e.g., Pinot gris) (Figure 1).

**Figure 2:**
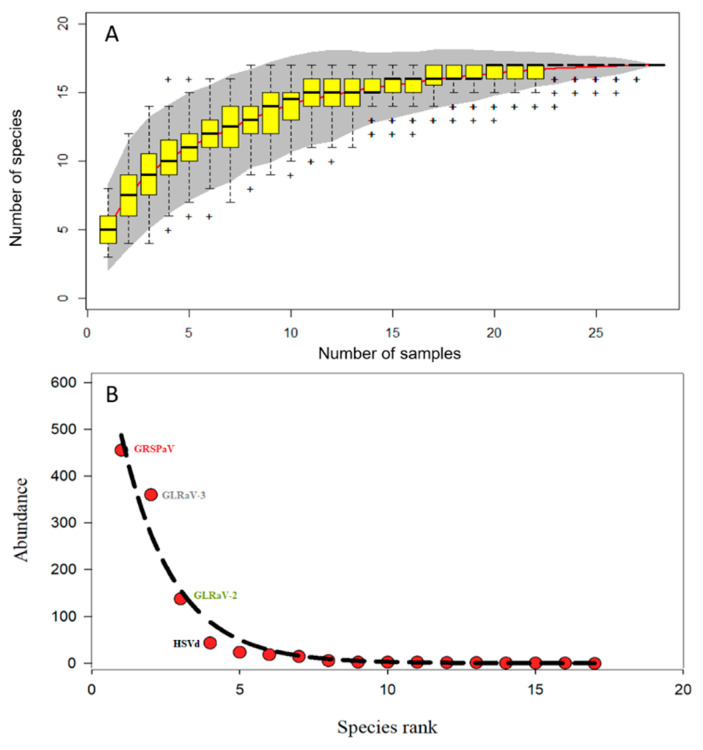
An accumulation curve of the viral population in grapevine plant samples (**A**). The red line represents the number of virus species as a function of number of samples collected. The gray area displays the standard deviation, and the yellow box plots show the species richness based on linear interpolation of random permutation. The virus rank abundance curve (**B**) displays the virus abundance as a function of virus species rank. The four virus and viroid species (grapevine Rupestris stem pitting-associated virus (GRSPaV) GLRaV-3, GLRaV-2, hop stunt viroid (HSVd)) in terms of abundance are shown. R packages vegan, gridExtra, ggplot2 and ade4 were used (see materials and methods section for more details).

**Figure 3:**
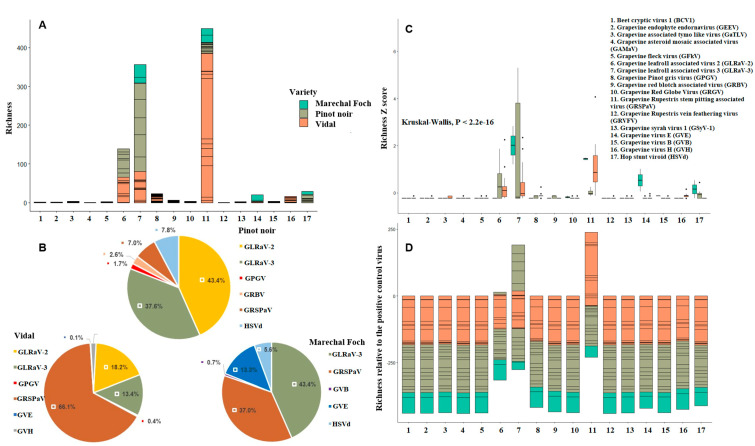
Virus richness (**A**), virus relative abundance (**B**), virus richness Z score (**C**) and virus richness relative to the richness of the positive control (*Phaseolus vulgaris endorvirus1* (PvEV1)) (**D**), sorted by the three major grapevine cultivars that constituted 93% of the total plants sampled (Marechal Foch, Pinot noir and Vidal). R package ggplot2 was used (see materials and methods section for details on how richness and richness Z score were calculated).

Over 2,228,600 reads were obtained with a percentage of mapped viral reads ranging between 3.12% and 19.50% of the total number of reads. The viral communities were relatively well-sampled across the three sampling sites. The plateau in terms of number of virus species was detected after a cumulus of 17 samples, and 60% of the viral species were detected after a cumulus of five samples, suggesting that many of the viral species were present in the majority of sampled plants (Figure 2A). The three most abundant virus species in the leafroll-infected samples were grapevine Ruspestris stem pitting virus (GRSPaV), grapevine leafroll-associated virus 3 (GLRaV-3) and grapevine leafroll-associated virus 2 (GLRaV-2). Hop stunt viroid (HSVd) was the fourth most abundant species across the grapevine samples (Figure 2B). Using the dsRNA sequencing method, it was possible to detect RNA and DNA viruses and viroids. Except for GLRaV-3, all the viruses and viroids reported in Figure 3 were detected for the first time in Quebec (Table 1). Even though three viruses (GRSPaV, GLR and GLRaV-2) and one viroid (HSVd) largely dominated the virome, their presence and abundance can be sorted by grapevine cultivars (Figure 3). In the hybrid cultivar Vidal, GRSPaV dominated the virome with 61% of the viral population, whereas GLRaV-3 and GLRaV-2 represented 13% and 18% of the viral population, respectively, and the other viruses occupied between 0.001% and 0.1% of the virome. Grapevine virus H was only present in the hybrid cultivar Vidal. In the cultivar Pinot noir (*Vitis vinifera*), GLRaV-2 dominated the virome with 43% of the viral population, whereas GRSPaV, GLRaV-3 and HSVd represented 7%, 38% and 8% of the viral population, respectively, and the other viruses occupied between 0.01% and 2.7% of the virome. In the cultivar Marechal Foch (hybrid cultivar), GLRaV-3 dominated the virome with 43% of the viral population, whereas GRSPaV, GVE and HSVd represented 37%, 13% and 5.6% of the viral population, respectively, and the other viruses occupied between 0.01% and 0.7% of the virome. The HSVd was only detected in the Pinot noir and Marechal Foch cultivars, whereas grapevine red blotch-associated virus (GRBV) was only detected in the Pinot noir cultivar (Figure 3A–D). When considering the species abundance relative to the abundance of the positive control virus (PvEV1) that was added to the samples, only GLRaV-2, GLRaV-3 and GRSPaV were more abundant than PvEV1. In Marechal Foch grapevine cultivar, none of the detected viruses seem to be more abundant than PvEV1 (Figure 3D).

**Figure 4:**
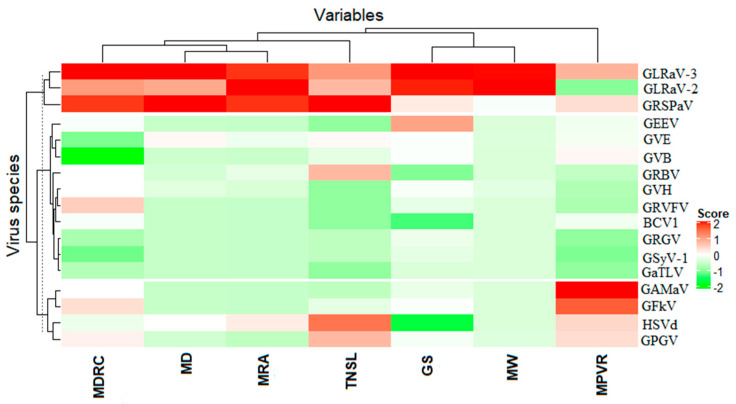
Heatmap displaying the characteristics and association between detected viruses and the mean proportion of viral reads that mapped for a given virus (MPVR), total number of symptomatic leaves (TNSL) associated with a given virus, mean depth (MD), mean depth relative to the depth of the positive control virus (MDRC), mean relative abundance (MRA), mean weight and genome size (GS). The pam function of the cluster package was used for partitioning the data into k cluster around Medoids (k-Medoids, (k = 3)) because it is less sensitive to outliers compared to k-means [33]. R packages ComplexHeatmap, circlize, colorspace, dendextend, cluster and GetoptLong were used (see materials and methods section for more details). For virus names and abbreviations, please see Table 1.

The optimal number of clusters based on independent variables (MPVR, MW, TNSL, MDRC, MD, MRA and GS) used as inputs was 2. One cluster was composed by GLRaV-3, GLRaV-2 and GRSPaV, and the other consisted of the remaining viruses. However, the latter cluster can be divided into two subclusters, one subcluster composed of grapevine asteroid mosaic virus (GAMaV), grapevine Fleck virus (GFkV), HSVd and grapevine pinot gris virus (GPGV) and a second composed of the rest of the viruses (Figure 4). When we collapsed the variables to have two dimensions using discriminant principal component analysis, the viral population was divided into four groups (GRSPaV, GLRaV-3, GLRaV-2 and the rest of the viruses) (Figure S1). Viruses and viroids that were associated with the highest number of plants expressing symptoms (variable TNSL) were GRSPaV, HSVd, GLRaV-3, GPG, GRBV and GLRaV-2 in gradient order (Figure 4). However, this association may be random and not directly linked to the symptoms because many NGS studies described GRSPaV as one of the viruses of the background virome. Nevertheless, the analysis of the association between virus and leafroll symptom development will bring more insights.

**Figure 5:**
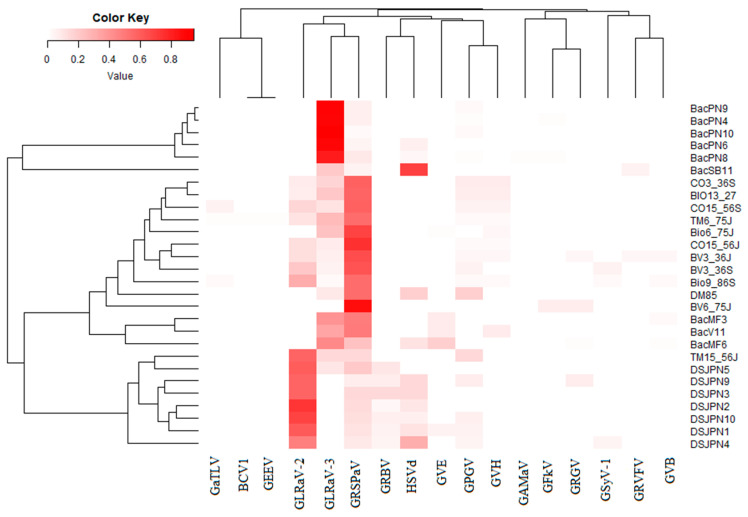
Heatmap of hierarchical clustering of grapevine virome composition profiles represented by the normalized relative abundance per grapevine sample. Heatmap color (white to dark red) displays the row-scaled relative abundance of each virus across all samples. The Bray–Curtis dissimilarity matrix and average linkage distance were used. R packages Vegan, Heatplus, gplots, RcolorBrewer and readxl were used (see materials and methods section for more details).

The viral signature (virome composition) was clustered by sampling site (Figure 5). Sample names starting with “Bac” were from the first commercial vineyard (Hem), those started with “DSJ” were from the second commercial vineyard (SJM), and the rest of the samples were from the experimental farm (Fr, see Plant Material section). Samples from the same site were in the same cluster, except for three samples from Hem vineyard, and the grapevine cultivar type seems to have no impact on it (Figure 5). The virome in the Hem and SJM commercial vineyards was dominated by GLRaV-3 and GLRaV-2, respectively, whereas it was dominated by GRSPaV at the experimental vineyard, except for four samples (Figure 5). The GRBV was only detected in the SJM commercial vineyard, and the new grapevine-associated tymo-like virus (GaTLV) was only detected in the Fr vineyard, whereas the grapevine asteroid mosaic-associated virus (GAMaV) was only detected in the Hem commercial vineyard (Figure 5, Table 1).

### 3.2. Association between Viruses and Virus-Like Symptom Development

**Figure 6:**
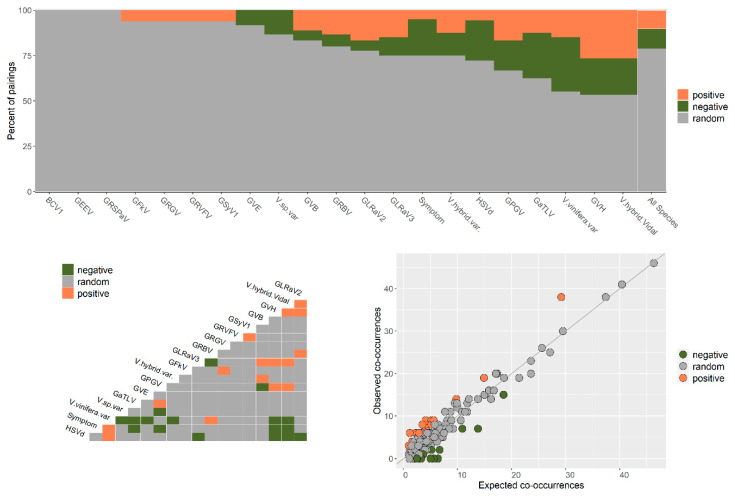
The percent of total pairings for virus species, leafroll-like symptoms and grapevine cultivars that are positive, negative and random (boxplot top graph). The bar outline in white shows the assemblage-wide percentages. A heatmap of the positive, negative and random associations (bottom-left graph) displays the probabilistic co-occurrence that observed frequency of co-occurrence between two events (e.g., virus, viroids or symptoms presence in a given sample) is significantly greater (large) than expected (meaning a positive association), significantly less (small) than expected (meaning negative association), or not significantly different and approximately equal to expectation (meaning random association)). Events are positioned to indicate the columns and the rows that represent their pairwise relationships. Graphic displaying the scatter plot of observed versus expected co-occurrence (bottom-right graph). Each event pair in the analysis is represented by a circle colored based on whether it was classified as positive (orange), negative (dark green) or random (gray). Any event pairs that were expected to share less than one sampling unit were removed from the analysis. R package co-occur was used (see materials and methods section for more details). See Table 1 for virus names and abbreviations.

The co-occurrence analysis removed 75 pairs (32.7%) that had an expected co-occurrence <1, and 156 pairs of combination were analyzed. Sixteen positive associations, 17 negative associations and 123 random associations were found (Table 2). Only those significant of the most important associations were displayed, and the events that did not have sufficient occurrence data were removed (Figure 6). Grapevine leafroll symptom expression had a significant positive association with the cultivar *Vitis vinifera* (Pgt < 0.00000) and a significant negative association with the cultivar Vidal (Hybrid) (Plt < 0.00000) (Table 2, Figure 6, Table S1). Presence of GLRaV species (GLRaV-2 and GLRaV-3) was randomly associated with symptom development. When the analyses of co-occurrence were done without the cultivar Vidal, the association between GLD species and symptom expression was still random, and grapevine leafroll-associated virus 3 (GLRaV-3) was significantly and negatively associated with hop stunt viroid (Pgt < 0.00050) and grapevine red blotch-associated virus (GRBV). However, the number of plants that were infected with GRBV was low (9), and therefore the negative association between GRBV and GLRaV-3 was considered as weak (Table 2, Figure 6).

### 3.3. The Genetic Diversity of Viruses and Viroids Detected for the First Time in Quebec

**Figure 7:**
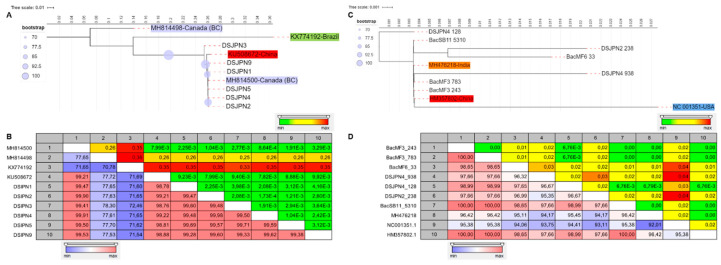
Maximum likelihood phylogenetic tree constructed from recovered consensus partial sequences of (**A**) grapevine leafroll-associated virus 2 (GLRaV-2) and full genome of (**C**) hop stunt viroid (HSVd). Neighbor-joining construction method with 1000 bootstrap replicates was used. Branch length represents phylogenetic distances determined with distance matrices of nucleotide sequences. Purple dots above critical branches are significant bootstrap values (>70%). GenBank accession number of fully sequenced genomes from different countries are highlighted for reference. Isolates from this study are represented and are not highlighted. The percent of identity (purple-red scale) and Jukes–Cantor distance (yellow-red scale) are presented for each pair of sequences of (**B**) GLRaV-2 and (**D**) HSVd. The percent of identity is the proportion of identical residues in the alignment positions to overlapping alignment positions between the two sequences. The distance was calculated using the Jukes–Cantor distance between the two sequences and is the proportion between identical and overlapping alignment position between two sequences. R package phylogram and the interactive tree of life platform were used to generate the tree (see the materials and methods section for more details).

**Figure 8:**
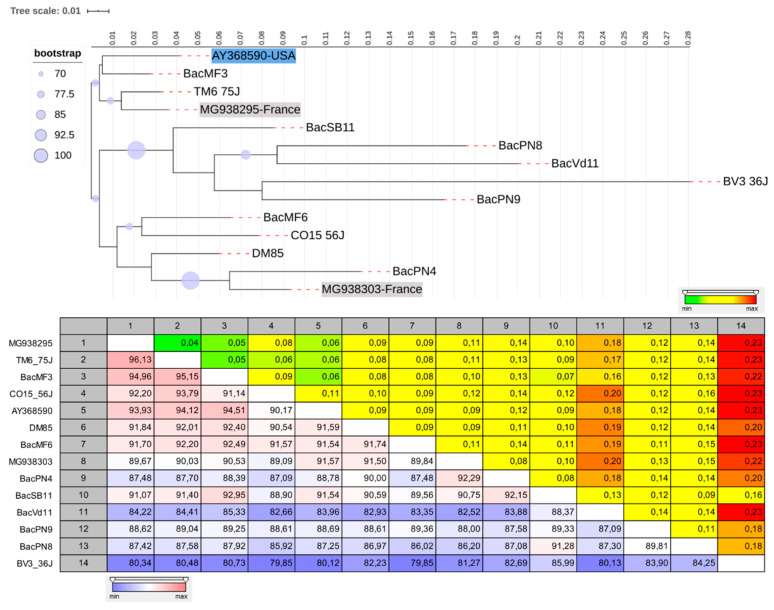
Maximum likelihood phylogenetic tree constructed from recovered consensus partial sequences of grapevine Ruspestris stem pitting-associated virus *(*GRSPaV*)*. Neighbor-joining construction method with 1000 bootstrap replicates was used. Branch length represents phylogenetic distances determined with distances matrices of nucleotide sequences. Purple dots above critical branches are significant bootstrap values (>70%). GenBank accession numbers of fully sequenced genomes from different countries are highlighted for reference. Isolates from this study are represented and are not highlighted. The percent of identity (purple-red scale) and Jukes–Cantor distance (yellow-red scale) are represented for each pair of sequences of GRSPaV. The percent of identity is the proportion of identical residues in the alignment positions to overlapping alignment positions between the two sequences. The distance was calculated using the Jukes–Cantor distance between the two sequences and is the proportion between identical and overlapping alignment position between two sequences. R package phylogram and the interactive tree of life platform was used to generate the tree (see the materials and methods section for more details).

Among all viruses that were detected, it was possible to recover consensus sequences for GLRaV-2, HSVd and GRSPaV phylogenetic analyses, and these sequences were deposited in GenBank (MT899925-MT899930, MT769768-MT769774, MT832848-MT832891 and MT855968-MT855978). For GLRaV-2, partial sequences (5788 bp) from six samples (Table S2), all from the cultivar Pinot noir, were recovered and used for the phylogenetic analysis. Four corresponding sequences from full genomes of GLRaV-2 from China, Brazil and Canada (BC) available in GenBank were aligned and used to construct a phylogenetic tree. All our isolates clustered in the same group with isolates originating from China and BC (Canada). Within this cluster, two subgroups were supported by a significant bootstrap value >70%. Comparative analysis revealed nucleotide (NT) identities in the range of 98.78% to 99.98% between our isolates and those from BC (Canada, MH814500) and China (KU508672). Our isolates were dissimilar to the isolates from Brazil (KX774192) and BC (Canada, MH814498) in terms of NT identity and Jukes–Cantor distance (70.78% to 78.30% NT identity, Figure 7A,B). For HSVd, seven whole genomes (297–299 bp) from five samples were used for the phylogenetic analysis. Three full genomes of HSVd from USA, China and India retrieved from GenBank were added for the alignment and the construction of the phylogenetic tree (Figure 7C). The phylogenetic relationship within HSVd isolates could not be determined due to the low bootstrap support for the clustering (Figure 7C). Comparative analysis revealed that HSVd sequences were highly similar to the isolate from China (HM357802) in terms of NT identity and Jukes–Cantor distance (97.66% to 100% NT identity, Figure 7D) and slightly dissimilar to the HSVd isolate from citrus in USA from 1988 (NC001351). Overall, our HSVd full genomes were highly similar to isolates from China, India and USA (92.01% to 100% NT identity) (Figure 7C,D). For GRSPaV, a total of 14 sequences, 11 from our isolates (5244 bp) (Table S3) and three corresponding sequences from GRSPaV full genome (one from USA and two from France) obtained from GenBank, were aligned and used to construct a phylogenetic tree (Figure 8). Two main well-supported GRSPaV phylogenetic groups were detected in the analysis of our isolates. Most of our isolates grouped into distant large subclusters. The first cluster was closely related (94.96% to 96.13% NT identity) to the GenBank sequences of GRSPaV from France (MG938295) and USA (AY368590) (Figure 8). The second cluster was phylogenetically diverse (79.85% to 92.95% NT identity) with some isolates (BacPN4, DM85, BacMF6 and Co15_56J) closely related (89.84% to 92.29% NT identity) to the GenBank sequence of GRSPaV isolate from France (MG938303). Presence of any GRSPaV phylo-groups was not associated with grapevine cultivars (Figure 8). Overall, all isolates of GRSPaV were in the same Clade 1 with recombinant genomes of isolates from France (MG938295 and MG938303) and USA (AY368590).

## 4. Discussion

Growers’ increasing experience and expertise in northern viticulture and climate change represent an opportunity for major change in Quebec’s grapevine industry by allowing a shift from traditionally cultivated hybrid cultivars (e.g., interspecific hybrids Vidal) into cultivars with lower cold hardiness and disease tolerance, such as *Vitis vinifera* (Pinot noir). However, this situation comes with potential risk including grapevine leafroll disease (GLD), one of the most economically damaging grapevine diseases. Considering that the disease is latent in some grapevine hybrid cultivars (e.g., Vidal) and that these cultivars will continue to be grown, they can serve as a virus reservoir [14]. In this study, we characterized the virome of the interspecific hybrid cultivars and compared it to the virome of a *V. vinifera* cultivar to guide grapevine producers during the transition.

Grapevine leafroll-infected plants had a diverse virome in terms of composition, abundance and richness. Three viruses (GRSPaV, GLRaV-3 and GLRaV-2) and one viroid (HSVd) largely dominated the overall virome. However, their presence and abundance can be sorted by grapevine cultivars. Indeed, in the hybrid cultivar Vidal, GRSPaV dominated the virome with 61% of the viral population, and GLRaV-3 and GLRaV-2 represented 13% and 18% of the viral population, respectively, whereas in the cultivar Pinot noir (*Vitis vinifera*), GLRaV-2 dominated the virome with 43% of the viral population, and GRSPaV, GLRaV-3 and HSVd represented 7%, 38% and 8% of the viral population, respectively. The virome composition was clustered by sampling site and not by grapevine cultivars. These results suggested that GLRaV-2 and GLRaV-3 were widely distributed in Quebec and in the cultivar Vidal even though the relative abundance of these viruses was lower in Vidal than in Pinot noir. Therefore, Vidal cultivar could serve as a virus reservoir and play a key epidemiological role during the transition. In addition, Vidal cultivar did not express any notable visual symptoms despite the presence of both GLRaV-2 and GLRaV-3. Indeed, leafroll symptom expression had a significant positive association with the cultivar *Vitis vinifera* and a significant negative association with cultivar Vidal (*Vitis* sp. Hybrid). These results are supported by Beuve et al. [9] and Kovacs et al. [14], who noticed strong symptom expression associated with grapevine cultivar Pinot noir and that the grapevine leafroll viruses are latent in Vidal cultivar. Therefore, from a strictly GLD disease management decision-making standpoint, Vidal should be classified with cultivars such as Thompson Seedless and Sauvignon Blanc, in which GLD is nearly impossible to detect visually [41]. When establishing new cultivars (e.g., *Vitis vinifera*), growers cannot rely only on clean planting materials derived from virus-tested stocks. They should also test their vineyards for at least the presence of GLR (2 and 3), and the testing should particularly include the symptomless Vidal cultivar. In vineyards where it is not possible to randomly test enough Vidal plants, managers should consider maximizing the distance between new plantings and the existing grapevine plants to reduce potential GLD spread.

Most of the virus and viroid species detected, including GRSPaV, HSVd, grapevine syrah virus 1 (GSyV-1) grapevine fleck virus and grapevine Red Globe, can be considered as part of the “background” virome. These background viruses and viroids are not primarily critical in the expression of virus-like symptoms [42]. Indeed, the symptom expressions are believed to be associated with additional infection of rarely-damaging viruses (GLRaV-3, GLRaV-2, grapevine pinot gris virus, grapevine red blotch, etc.) that disrupt the existing virome, leading to the expression of symptoms [42]. Our data show that viruses and viroids associated with the highest number of plants expressing leafroll symptoms (variables TNSL) were GRSPaV, HSVd, GLRaV-3, GPG, GRBV and GLRaV-2, in gradient order. However, co-occurrence analysis revealed that the presence of GLR species (GLRaV-2 and GLRaV-3) was randomly associated with symptom development. This conclusion derived from analysis of 1452 points of comparison resulting from compilation of our data and data from five other published studies. In white grapevine cultivars such as Vidal, the symptoms are often subtle and difficult to diagnose, which can induce a bias in the co-occurrence analysis. However, even when the co-occurrence analyses were conducted without Vidal cultivar, the association between GLD virus species and symptom expression was still random. A possible explanation of this unexpected result may be that the link between GLR viruses (2 and 3) and the symptoms that have been demonstrated in many published studies is more likely related to the virus titer in a given plant than its presence or absence. Indeed, co-occurrence analyses are based on presence and absence and do not consider the virus titer. Hence, this is not an evidence of quantitative interaction between GLR viruses and symptom expression [43]. Monis and Bestwick demonstrated that symptomatic leafroll-infected leaves of grapevine had high titers of the virus and while undetectable virus titers was obtained from no leafroll symptoms samples [44].

Because symptoms result from complex abiotic and biotic interactions [8], the role of abiotic factors (e.g., weather) and the different genetic variants of GLR and other viruses and viroids that were detected may also explain this unexpected result. In fact, association between GLRaV-2 and symptoms were linked with the phylogenetic clustering of the genetic variants [45], and Thompson et al. [46] discovered a novel genetic variant of GLRaV-3 that induces no foliar symptoms. However, we did not have enough consensus sequences of GLRaV-3 to study the phylogenetic diversity of GLRaV-3 strains associated with our samples. The phylogenetic analysis of GLRaV-2 strains associated with our samples revealed a single monophyletic group that is highly similar to some isolates from BC and also significantly dissimilar to others isolates from BC. As in BC, this monophyletic group was associated with the cultivar Pinot noir, which indicated possible infection via planting materials [16]. However, as highlighted by Poojari et al. [16], there is a need to understand the impact of GLRaV-2 genetic variants in Canadian vineyards. In summary, more research is needed to shed light on GLD symptom expression resulting from the interaction of different genetic variants of GLR (2 and 3) and grapevine cultivars. Moreover, the role of genetic variability among strains of the most abundant viruses/viroids of the background virome, such as GRSPaV and HSVd, should be studied. Indeed, our isolates of GRSPaV were grouped into distant large clusters and were all classified in the same clade (clade 1) with other recombinant genomes from France [47]. Until today, no published studies have shown a relationship between strains from the four main clades of GRSPaV and symptom development even though it is known that some strains of GRSPaV can cause symptoms on certain grapevine rootstock genotype (*Vitis Rupestris* cv. St. George) [48]. Implication of HSVd in symptom development, which is not yet proven, cannot be related to the genetic variability among our HSVd strains because our HSVd sequences were highly similar and no significant dissimilarities of our HSVd full genomes were observed when compared to known grapevine HSVd. Nevertheless, as these two members of the background virome are frequently observed in grapevine-infected plants [9,42,49], more studies are needed to understand the corpus of these viruses and their potential role in symptom expression.

## 5. Conclusions

This study highlights the complexity of the grapevine virome associated with leafroll-infected plants of different cultivars. Independently of the grapevine cultivar, the plants were infected by multiple viral and viroid species and different variants of the same virus. These results are supported by Beuve et al. However, these authors described this complex virome only on the cultivar Pinot noir [9]. The symptomless hybrid cultivar Vidal presents a complex virome which, in contrast, was dominated by grapevine Rupestris stem pitting-associated virus despite the presence of grapevine leafroll disease (GLD) virus species. Therefore, removing and destroying of symptomatic grapevines and possibly vines immediately adjacent to those symptomatic grapevines, which is proven to be efficient to control GLD spread [8,50,51], will not be effective in vineyards where Vidal grapevines are grown because this latter cultivar will hamper the management efforts. During the transition and establishment of new and less winter hardy cultivars such as *Vitis vinifera* (e.g., Pinot noir) in Quebec, growers cannot rely only on certified material derived from virus-tested stocks, they also need to periodically test their vineyards for at least the presence of GLRaV (2 and 3), and the testing should particularly include the symptomless Vidal cultivar.

## Figures and Tables

**Table 1:** Detection and description of viruses detected by dsRNA sequencing and confirmed by PCR reaction from composite samples of grapevine leaves collected at the two commercial vineyards (Hemmingford (Hem) and Saint-Jacques-le-Mineur (SJM)) and the experimental vineyard (Fr).

Virus Name	Species (ICTV) ^a^	Abbreviation	NGS ^b^	PCR ^c^	Sampling Sites		
					Fr	Hem	SJM
Beet cryptic virus 1	*Beet cryptic virus 1*	BCV1	+	NT	+	-	-
Grapevine endophyte endornavirus	*Grapevine endophyte alphaendornavirus*	GEEV	+	NT	+	-	-
Grapevine associated tymo like virus	Not been approved yet	GaTLV	+	NT	+	-	-
Grapevine asteroid mosaic associated virus	Not been approved yet	GAMaV	+	NT	-	+	-
Grapevine fleck virus	*Grapevine fleck virus*	GFkV	+	+	+	+	-
Grapevine leafroll associated virus 2	*Grapevine leafroll-associated virus 2*	GLRaV-2	+	+	+	+	+
Grapevine leafroll associated virus 3	*Grapevine leafroll-associated virus 3*	GLRaV-3	+	+	+	+	+
Grapevine Pinot gris virus	*Grapevine Pinot gris virus*	GPGV	+	+	+	+	+
Grapevine red blotch associated virus	*Grapevine red blotch virus*	GRBV	+	+	-	-	+
Grapevine Red Globe Virus	*Grapevine red globe virus*	GRGV	+	NT	+	-	+
Grapevine Rupestris stem pitting associated virus	*Grapevine rupestris stem pitting-associated virus*	GRSPaV	+	NT	+	+	+
Grapevine Rupestris vein feathering virus	Not been approved yet	GRVFV	+	NT	+	+	-
Grapevine syrah virus 1	Not been approved yet	GSyV-1	+	+	+	-	+
Grapevine virus E	*Grapevine virus E*	GVE	+	NT	-	+	+
Grapevine virus B	*Grapevine virus B*	GVB	+	NT	+	+	-
Grapevine virus H	Not been approved yet	GVH	+	NT	+	+	-
Hop stunt viroid	*Hop stunt viroid*	HSVd	+	+	-	+	+

^a^ ICTV virus species names following the guidelines of the international committee on taxonomy of viruses (ICTV), ^b^ next generation sequencing technology using dsRNA extraction method, ^c^ polymerase chain reaction; NT for not tested.

**Table 2:** Co-occurrence table displaying the events that are significantly associated with leafroll-like symptom development and presence of grapevine leafroll-associated virus 3 (GLRaV-3).

	Event ^a^	Ev_Inc ^b^	Obs_Cooccur ^c^	Prob_Cooccur ^d^	Exp_Cooccur ^e^	p_lt ^f^	p_gt ^g^
Symptom expression	Hybrid Vidal	9	0	0.097	6.4	0.00000	1.00000
	*V. vinifera* cvs	41	38	0.442	29.2	1.00000	0.00000
	GaTLV	4	0	0.043	2.8	0.00538	1.00000
	GPGV	26	15	0.281	18.5	0.04747	0.98688
	GVH	9	0	0.097	6.4	0.00000	1.00000
GLRaV-3 presence	GRBV	9	2	0.079	5.2	0.02587	0.99662
	GVB	17	14	0.148	9.8	0.99742	0.01517
	GVH	9	9	0.079	5.2	1.00000	0.00440
	HSVd	24	7	0.209	13.8	0.00050	0.99994
	Hybrid Vidal	9	8	0.079	5.2	0.99560	0.04140

^a^ Represents virus presence, symptom presence in a given sample or a given variety; ^b^ represents number of sampling sites where a given event occurs; ^c^ represents observed number of sampling sites having two given events; ^d^ represents probability that the two given events occur at a sampling site; ^e^ represents expected number of sampling sites with the two given events; ^f^ represents probability that the two events would co-occur at a frequency less than the observed number of co-occurrence sampling sites if the two events were distributed randomly; ^g^ represents probability of co-occurrence at a frequency greater than the observed frequency.

## Supplementary Materials

The following are available online at https://www.mdpi.com/1999-4915/12/10/1142/s1, Figure S1: Discriminant principal component analysis of the virome and association between detected viruses and the mean proportion of viral reads that mapped for a given virus (MPVR), total number of symptomatic leaves (TNSL) associated with a given virus, mean depth (MD), mean depth relative to the depth of the positive control virus (MDRC), mean relative abundance (MRA), mean weight, and genome size (GS). Showing groups of species that induce similar response. Table S1: Co-occurrence table displaying association between all events. Table S2: GenBank accession numbers of Grapevine leafroll-associated virus 2 and their nucleotide positions of sequences used for generation of concatenated sequences in this study. Table S3: GenBank accession numbers of Grapevine Rupestris stem pitting-associated virus and their nucleotide positions of sequences used for generation of concatenated sequences in this study.

## References

[1] Statistic Canada Table 32-10-0364-01 Area, Production and Farm Gate Value of Marketed Fruits.

[2] Martelli G.P. (2014). Directory of virus and virus-like diseases of the grapevine and their agents. J. Plant Pathol..

[3] Fuchs M. (2020). Grapevine viruses: A multitude of diverse species with simple but overall poorly adopted management solutions in the vineyard. J. Plant Pathol..

[4] Alabi O.J., Casassa L.F., Gutha L.R., Larsen R.C., Henick-Kling T., Harbertson J.F., Naidu R.A. (2016). Impacts of Grapevine Leafroll Disease on Fruit Yield and Grape and Wine Chemistry in a Wine Grape (*Vitis vinifera* L.) Cultivar. PLoS ONE.

[5] Atallah S.S., Gomez M.I., Fuchs M.F., Martinson T.E. (2012). Economic Impact of Grapevine Leafroll Disease on Vitis vinifera cv. Cabernet franc in Finger Lakes Vineyards of New York. Am. J. Enol. Vitic..

[6] Martelli G.P., Boudon-Padieu E. (2006). Directory of infectious diseases of grapevines. International centre for advanced mediterranean agronomic studies. Options Méditerr. Ser. B Stud. Res..

[7] Poojari S., Alabi O.J., Fofanov V.Y., Naidu R.A. (2013). A leafhopper-transmissible DNA virus with novel evolutionary lineage in the family geminiviridae implicated in grapevine redleaf disease by next-generation sequencing. PLoS ONE.

[8] Almeida R.P., Daane K.M., Bell V.A., Blaisdell G.K., Cooper M.L., Herrbach E., Pietersen G. (2013). Ecology and management of grapevine leafroll disease. Front. Microbiol..

[9] Beuve M., Hily J.M., Alliaume A., Reinbold C., Le Maguet J., Candresse T., Herrbach E., Lemaire O. (2018). A complex virome unveiled by deep sequencing analysis of RNAs from a French Pinot noir grapevine exhibiting strong leafroll symptoms. Arch. Virol..

[10] Fuchs M., Marsella-Herrick P., Loeb G.M., Martinson T.E., Hoch H.C. (2009). Diversity of ampeloviruses in mealybug and soft scale vectors and in grapevine hosts from leafroll-affected vineyards. Phytopathology.

[11] Golino D.A., Weber E., Sim S., Rowhani A. (2008). Leafroll disease is spreading rapidly in a Napa Valley vineyard. Calif. Agric..

[12] Le Maguet J., Beuve M., Herrbach E., Lemaire O. (2012). Transmission of six ampeloviruses and two vitiviruses to grapevine by Phenacoccus aceris. Phytopathology.

[13] Poojari S., Moreau D.L., Kahl D., Ritchie M., Ali S., Úrbez-Torres J.R. (2020). Disease incidence and genetic variability of economically important grapevine viruses in Nova Scotia. Can. J. Plant Pathol..

[14] Kovacs L.G., Hanami H., Fortenberry M., Kaps M.L. (2001). Latent infection by leafroll agent GLRaV-3 is linked to lower fruit quality in french-american hybrid grapevines Vidal blanc and St Vincent. Am J Enol Vitic..

[15] MacKenzie D.J., Johnson R.C., Warner C. (1996). Incidence of four important viral pathogens in Canadian vineyards. Plant Dis..

[16] Poojari S., Boule J., DeLury N., Lowery D.T., Rott M., Schmidt A.M., Urbez-Torres J.R. (2017). Epidemiology and Genetic Diversity of Grapevine Leafroll-Associated Viruses in British Columbia. Plant Dis..

[17] Poojari S., Lowery D.T., Rott M., Schmidt A.M., Úrbez-Torres J.R. (2017). Incidence, distribution and genetic diversity of Grapevine red blotch virus in British Columbia. Can. J. Plant Pathol..

[18] Xiao H., Shabanian M., Moore C., Li C., Meng B. (2018). Survey for major viruses in commercial Vitis vinifera wine grapes in Ontario. Virol. J..

[19] du Québec G. (2020). Profil Sectoriel de L’industrie Bioalimentaire au Québec.

[20] Gouvernement du Québec (2009). Profil Sectoriel de L’industrie Bioalimentaire au Québec.

[21] Xiao H., Li C., Al Rwahnih M., Dolja V., Meng B. (2019). Metagenomic Analysis of Riesling Grapevine Reveals a Complex Virome Including Two New and Divergent Variants of Grapevine leafroll-associated virus 3. Plant Dis..

[22] Ben Moussa I.E., Lemoyne P., Fall M.L. (2019). Virus and grapevine, unbreakable relationship: Biovigilance is more than require. Phytoprotection.

[23] Carisse O., Fall M.L., Vincent C. (2017). Using a biovigilance approach for pest and disease management in Quebec vineyards. Can. J. Plant Pathol..

[24] Alkowni R., Zhang Y.P., Rowhani A., Uyemoto J.K., Minafra A. (2011). Biological, molecular, and serological studies of a novel strain of grapevine leafroll-associated virus 2. Virus Genes.

[25] Vionnet L., De Vrieze M., Agnès D., Gfeller A., Lüthi A., L’Haridon F., Weisskopf L. (2018). Microbial life in the grapevine: What can we expect from the leaf microbiome?. OENO One.

[26] Xiao H., Kim W.S., Meng B. (2015). A highly effective and versatile technology for the isolation of RNAs from grapevines and other woody perennials for use in virus diagnostics. Virol. J..

[27] Poojari S., Alabi O.J., Okubara P.A., Naidu R.A. (2016). SYBR((R)) Green-based real-time quantitative reverse-transcription PCR for detection and discrimination of grapevine viruses. J. Virol. Methods.

[28] Kesanakurti P., Belton M., Saeed H., Rast H., Boyes I., Rott M. (2016). Screening for plant viruses by next generation sequencing using a modified double strand RNA extraction protocol with an internal amplification control. J. Virol. Methods.

[29] Bolger A.M., Lohse M., Usadel B. (2014). Trimmomatic: A flexible trimmer for Illumina sequence data. Bioinformatics.

[30] Rott M., Xiang Y., Boyes I., Belton M., Saeed H., Kesanakurti P., Hayes S., Lawrence T., Birch C., Bhagwat B. (2017). Application of Next Generation Sequencing for Diagnostic Testing of Tree Fruit Viruses and Viroids. Plant Dis..

[31] Ho T., Tzanetakis I.E. (2014). Development of a virus detection and discovery pipeline using next generation sequencing. Virology.

[32] Kembel S.W., Cahill J.F. (2011). Independent evolution of leaf and root traits within and among temperate grassland plant communities. PLoS ONE.

[33] Kaufman L., Rousseeuw P.J. (1990). Partitioning Around Medoids (Program PAM). An Introduction to Cluster Analysis.

[34] Veech J.A., Peres-Neto P. (2013). A probabilistic model for analysing species co-occurrence. Glob. Ecol. Biogeogr..

[35] Griffith D.M., Veech J.A., Marsh C.J. (2016). Cooccur: Probabilistic Species Co-Occurrence Analysis inR. J. Stat. Softw..

[36] Al Rwahnih M., Daubert S., Golino D., Islas C., Rowhani A. (2015). Comparison of Next-Generation Sequencing Versus Biological Indexing for the Optimal Detection of Viral Pathogens in Grapevine. Phytopathology.

[37] Eichmeier A., Kominkova M., Kominek P., Baranek M. (2016). Comprehensive Virus Detection Using Next Generation Sequencing in Grapevine Vascular Tissues of Plants Obtained from the Wine Regions of Bohemia and Moravia (Czech Republic). PLoS ONE.

[38] Fajardo T.V.M., Silva F.N., Eiras M., Nickel O. (2017). High-throughput sequencing applied for the identification of viruses infecting grapevines in Brazil and genetic variability analysis. Trop. Plant Pathol..

[39] Giampetruzzi A., Roumi V., Roberto R., Malossini U., Yoshikawa N., La Notte P., Terlizzi F., Credi R., Saldarelli P. (2012). A new grapevine virus discovered by deep sequencing of virus- and viroid-derived small RNAs in Cv Pinot gris. Virus Res..

[40] Letunic I., Bork P. (2019). Interactive Tree Of Life (iTOL) v4: Recent updates and new developments. Nucleic Acids Res..

[41] Maree H.J., Almeida R.P., Bester R., Chooi K.M., Cohen D., Dolja V.V., Fuchs M.F., Golino D.A., Jooste A.E., Martelli G.P. (2013). Grapevine leafroll-associated virus 3. Front. Microbiol..

[42] Saldarelli P., Giampetruzzi A., Maree H.J., Al Rwahnih M., Meng B., Martelli G.P., Golino D.A., Fuchs M. (2017). High-Throughput Sequencing: Advantages Beyond Virus Identification. Grapevine Viruses: Molecular Biology, Diagnostics and Management.

[43] Blanchet F.G., Cazelles K., Gravel D. (2020). Co-occurrence is not evidence of ecological interactions. Ecol. Lett..

[44] Monis J., Bestwick R.K. (1996). Detection and Localization of Grapevine Leafroll Associated Closteroviruses in Greenhouse and Tissue Culture Grown Plants. Am. J. Enol. Vitic..

[45] Bertazzon N., Borgo M., Vanin S., Angelini E. (2010). Genetic variability and pathological properties of Grapevine Leafroll-associated Virus 2 isolates. Eur. J. Plant Pathol..

[46] Thompson B.D., Dahan J., Lee J., Martin R.R., Karasev A.V. (2019). A Novel Genetic Variant of Grapevine leafroll-associated virus-3 (GLRaV-3) from Idaho Grapevines. Plant Dis..

[47] Hily J.M., Beuve M., Vigne E., Demangeat G., Candresse T., Lemaire O. (2018). A genome-wide diversity study of grapevine rupestris stem pitting-associated virus. Arch. Virol..

[48] Reynolds A.G., Lanterman W.S., Wardle D.A. (1997). Yield and Berry Composition of Five Vitis Cultivars as Affected by Rupestris Stem Pitting Virus. Am. J. Enol. Vitic..

[49] Hily J.M., Candresse T., Garcia S., Vigne E., Tanniere M., Komar V., Barnabe G., Alliaume A., Gilg S., Hommay G. (2018). High-Throughput Sequencing and the Viromic Study of Grapevine Leaves: From the Detection of Grapevine-Infecting Viruses to the Description of a New Environmental Tymovirales Member. Front. Microbiol..

[50] Cabaleiro C., Segura A. (2006). Temporal Analysis of Grapevine leafroll associated virus 3 Epidemics. Eur. J. Plant Pathol..

[51] Habili N., Nutter F.W. (1997). Temporal and Spatial Analysis of Grapevine Leafroll-Associated Virus 3 in Pinot noir Grapevines in Australia. Plant Dis..

