# Supplementary material for: A Diverse Virome of Leafroll-Infected Grapevine Unveiled by dsRNA Sequencing
Source: Viruses. 2020 Oct 8;12(10):1142. doi: 10.3390/v12101142 (PMC7599845; doi:10.3390/v12101142)
Supplement: Supplementary file 1 [file viruses-12-01142-s001.pdf]

viruses

Supplementary Material Figures

A Diverse Virome of Grapevine Leafroll-Infected Grapevine Leaves Unveiled by dsRNA Sequencing

Mamadou L. Fall [1,*], Dong Xu [1], Pierre Lemoyne [1], Issam E. Ben Moussa [1,2], Carole Beaulieu [2] and Odile Carisse [1]

1 Saint-Jean-sur-Richelieu Research and Development Centre, Agriculture and Agri-Food Canada, St-Jean-sur-Richelieu, Quebec, QC J3B 3E6, Canada; dong.xu@canada.ca (D.X.); pierre.lemoyne@canada.ca (P.L.); Issam.Eddine.Ben.Moussa@USherbrooke.ca (I.E.B.M.); odile.carisse@canada.ca (O.C.)
2 Département de Biologie, Université de Sherbrooke, Sherbrooke, QC J1K 2R1, Canada; Carole.Beaulieu@USherbrooke.ca
* Correspondence: mamadoulamine.fall@canada.ca; Tel.: +1-579-224-3024

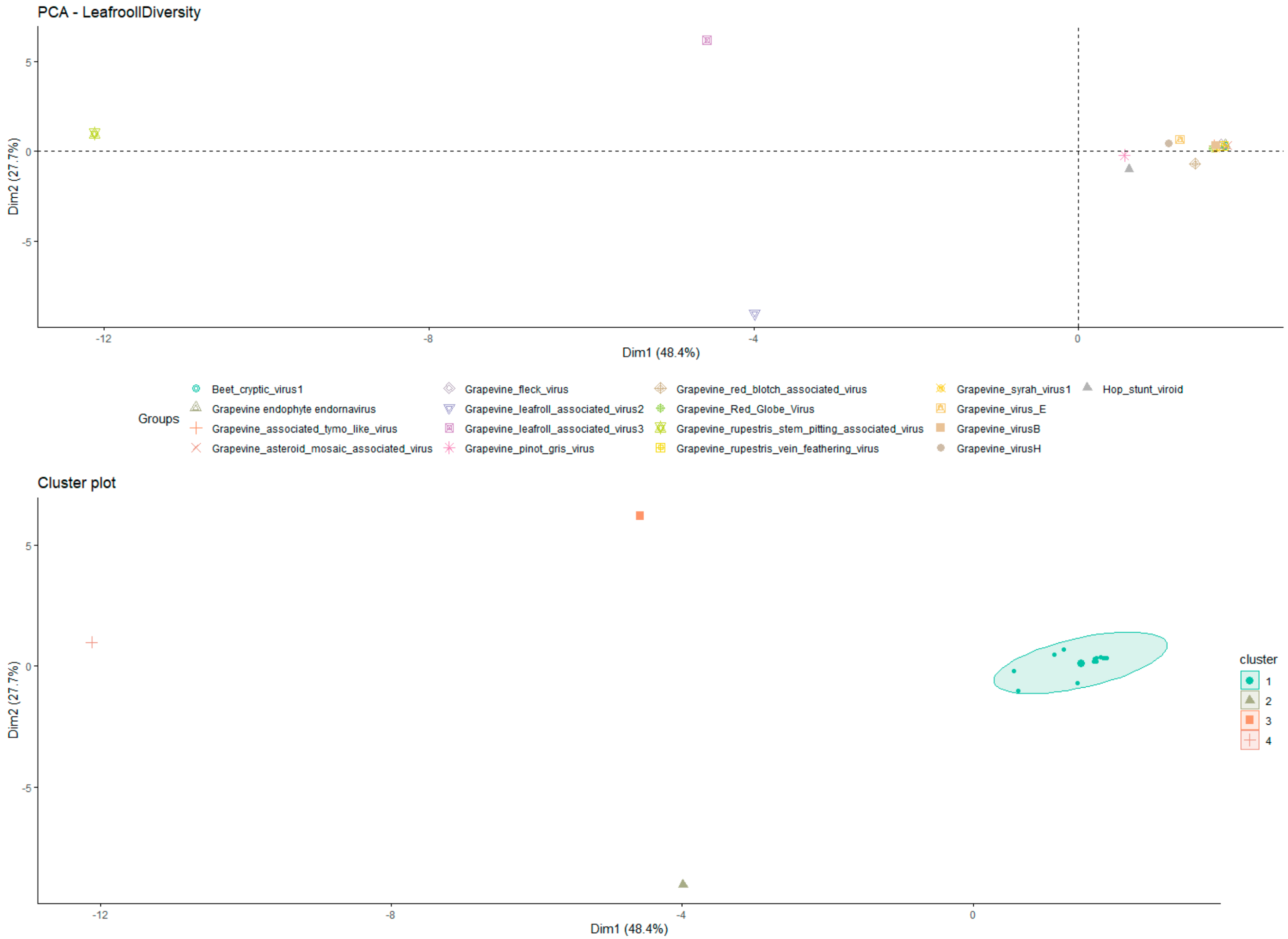

Figure S1. Discriminant principal component analysis of the virome and association between detected viruses and the mean proportion of viral read that mapped for a given virus (MPVR), total number of symptomatic leaf (TNSL) associated with a given virus, mean depth (MD), mean depth relative to the depth of the positive control virus (MDRC), mean relative abundance (MRA), the mean weight and genome size (GS). Showing groups of species that induce similar response.

Table S1. Co-occurence table displaying association between all events

Event_1	Event_2	Event1_inc	Event2_inc	obs_cooccur	prob_cooccur	exp_cooccur	p_lt	p_gt
BCV1	GLRaV3	2	38	2	0.017	1.2	1	0.32774
BCV1	GRSPaV	2	65	2	0.03	2	1	0.9697
BCV1	Symptom	2	47	0	0.022	1.4	0.07972	1
BCV1	V.vinifera.var	2	41	0	0.019	1.2	0.13986	1
GEEV	GLRaV3	2	38	2	0.017	1.2	1	0.32774
GEEV	GRSPaV	2	65	2	0.03	2	1	0.9697
GEEV	Symptom	2	47	0	0.022	1.4	0.07972	1
GEEV	V.vinifera.var	2	41	0	0.019	1.2	0.13986	1
GaTLV	GLRaV2	4	30	3	0.028	1.8	0.96198	0.24082
GaTLV	GLRaV3	4	38	4	0.035	2.3	1	0.10242
GaTLV	GPGV	4	26	4	0.024	1.6	1	0.02074
GaTLV	GRSPaV	4	65	4	0.06	3.9	1	0.93939
GaTLV	GVB	4	17	1	0.016	1	0.72855	0.70602
GaTLV	HSVd	4	24	0	0.022	1.5	0.1553	1
GaTLV	Symptom	4	47	0	0.043	2.8	0.00538	1
GaTLV	V.vinifera.var	4	41	0	0.038	2.5	0.01755	1
GFkV	GLRaV2	14	30	6	0.096	6.4	0.53488	0.69723
GFkV	GLRaV3	14	38	9	0.122	8.1	0.80874	0.39821
GFkV	GPGV	14	26	4	0.084	5.5	0.26914	0.89456
GFkV	GRBV	14	9	2	0.029	1.9	0.71294	0.61597
GFkV	GRGV	14	11	6	0.035	2.3	0.99907	0.0082
GFkV	GRSPaV	14	65	14	0.209	13.8	1	0.78788
GFkV	GRVFV	14	16	5	0.051	3.4	0.92672	0.21464
GFkV	GSyV-1	14	12	2	0.039	2.5	0.50614	0.7863
GFkV	GVE	14	6	0	0.019	1.3	0.22407	1
GFkV	GVB	14	17	5	0.055	3.6	0.90128	0.26295
GFkV	GVH	14	9	0	0.029	1.9	0.0994	1

GFkV	HSVd	14	24	3	0.077	5.1	0.15994	0.95198
GFkV	Symptom	14	47	12	0.151	10	0.96106	0.15436
GFkV	V.hybrid.Vidal	14	9	1	0.029	1.9	0.38403	0.9006
GFkV	V.vinifera.var	14	41	11	0.132	8.7	0.96324	0.13062
GFkV	V.sp.var	14	8	2	0.026	1.7	0.77968	0.54288
GLRaV2	GLRaV3	30	38	20	0.262	17.3	0.94735	0.13253
GLRaV2	GPGV	30	26	13	0.179	11.8	0.80258	0.36471
GLRaV2	GRBV	30	9	9	0.062	4.1	1	0.00039
GLRaV2	GRGV	30	11	6	0.076	5	0.84	0.36841
GLRaV2	GRSPaV	30	65	30	0.448	29.5	1	0.54545
GLRaV2	GRVFV	30	16	5	0.11	7.3	0.15329	0.9468
GLRaV2	GSyV-1	30	12	5	0.083	5.5	0.51412	0.72753
GLRaV2	GVE	30	6	1	0.041	2.7	0.14591	0.97856
GLRaV2	GVB	30	17	11	0.117	7.7	0.98378	0.05851
GLRaV2	GVH	30	9	7	0.062	4.1	0.99392	0.04073
GLRaV2	HSVd	30	24	7	0.165	10.9	0.03898	0.98905
GLRaV2	Symptom	30	47	19	0.324	21.4	0.15451	0.94103
GLRaV2	V.hybrid.Vidal	30	9	8	0.062	4.1	0.99961	0.00608
GLRaV2	V.vinifera.var	30	41	19	0.282	18.6	0.66927	0.52841
GLRaV2	V.hybrid.var.	30	4	0	0.028	1.8	0.08173	1
GLRaV2	V.sp.var	30	8	2	0.055	3.6	0.19639	0.95113
GLRaV3	GPGV	38	26	15	0.227	15	0.60536	0.59547
GLRaV3	GRBV	38	9	2	0.079	5.2	0.02587	0.99662
GLRaV3	GRGV	38	11	7	0.096	6.3	0.78003	0.46055
GLRaV3	GRSPaV	38	65	38	0.567	37.4	1	0.42424
GLRaV3	GRVFV	38	16	7	0.14	9.2	0.15988	0.94202
GLRaV3	GSyV-1	38	12	5	0.105	6.9	0.18121	0.93951
GLRaV3	GVE	38	6	5	0.052	3.5	0.96962	0.18507
GLRaV3	GVB	38	17	14	0.148	9.8	0.99742	0.01517
GLRaV3	GVH	38	9	9	0.079	5.2	1	0.0044

GLRaV3	HSVd	38	24	7	0.209	13.8	5e-04	0.99994
GLRaV3	Symptom	38	47	25	0.41	27.1	0.19603	0.92187
GLRaV3	V.hybrid.Vidal	38	9	8	0.079	5.2	0.9956	0.0414
GLRaV3	V.vinifera.var	38	41	20	0.358	23.6	0.05437	0.98352
GLRaV3	V.hybrid.var.	38	4	2	0.035	2.3	0.56984	0.79886
GLRaV3	V.sp.var	38	8	4	0.07	4.6	0.46198	0.80116
GPGV	GRBV	26	9	4	0.054	3.5	0.7605	0.50527
GPGV	GRGV	26	11	2	0.066	4.3	0.10541	0.97733
GPGV	GRSPaV	26	65	26	0.388	25.6	1	0.60606
GPGV	GRVFV	26	16	6	0.096	6.3	0.55024	0.6779
GPGV	GSyV-1	26	12	6	0.072	4.7	0.8759	0.30361
GPGV	GVE	26	6	2	0.036	2.4	0.55744	0.76946
GPGV	GVB	26	17	2	0.101	6.7	0.00588	0.99932
GPGV	GVH	26	9	8	0.054	3.5	0.99992	0.00177
GPGV	HSVd	26	24	13	0.143	9.5	0.98275	0.05576
GPGV	Symptom	26	47	15	0.281	18.5	0.04747	0.98688
GPGV	V.hybrid.Vidal	26	9	8	0.054	3.5	0.99992	0.00177
GPGV	V.vinifera.var	26	41	14	0.245	16.2	0.19534	0.91544
GPGV	V.hybrid.var.	26	4	1	0.024	1.6	0.48323	0.8732
GPGV	V.sp.var	26	8	0	0.048	3.2	0.01339	1
GRBV	GRGV	9	11	1	0.023	1.5	0.53362	0.82822
GRBV	GRSPaV	9	65	9	0.134	8.9	1	0.86364
GRBV	GRVFV	9	16	1	0.033	2.2	0.29976	0.93231
GRBV	GSyV-1	9	12	1	0.025	1.6	0.48099	0.85633
GRBV	GVB	9	17	2	0.035	2.3	0.57825	0.73737
GRBV	GVH	9	9	0	0.019	1.2	0.24305	1
GRBV	HSVd	9	24	6	0.05	3.3	0.99108	0.05067
GRBV	Symptom	9	47	8	0.097	6.4	0.96319	0.19823
GRBV	V.hybrid.Vidal	9	9	0	0.019	1.2	0.24305	1
GRBV	V.vinifera.var	9	41	9	0.085	5.6	1	0.00947

GRBV	V.sp.var	9	8	0	0.017	1.1	0.2877	1
GRGV	GRSPaV	11	65	11	0.164	10.8	1	0.83333
GRGV	GRVFV	11	16	5	0.04	2.7	0.9815	0.08312
GRGV	GSyV-1	11	12	4	0.03	2	0.97799	0.10363
GRGV	GVE	11	6	1	0.015	1	0.74023	0.68094
GRGV	GVB	11	17	5	0.043	2.8	0.97375	0.10682
GRGV	GVH	11	9	1	0.023	1.5	0.53362	0.82822
GRGV	HSVd	11	24	2	0.061	4	0.15144	0.96314
GRGV	Symptom	11	47	8	0.119	7.8	0.67525	0.60845
GRGV	V.hybrid.Vidal	11	9	2	0.023	1.5	0.83516	0.46638
GRGV	V.vinifera.var	11	41	7	0.104	6.8	0.66851	0.59627
GRGV	V.sp.var	11	8	1	0.02	1.3	0.60063	0.78801
GRSPaV	GRVFV	65	16	16	0.239	15.8	1	0.75758
GRSPaV	GSyV-1	65	12	11	0.179	11.8	0.18182	1
GRSPaV	GVE	65	6	6	0.09	5.9	1	0.90909
GRSPaV	GVB	65	17	16	0.254	16.7	0.25758	1
GRSPaV	GVH	65	9	9	0.134	8.9	1	0.86364
GRSPaV	HSVd	65	24	23	0.358	23.6	0.36364	1
GRSPaV	Symptom	65	47	46	0.701	46.3	0.71212	1
GRSPaV	V.hybrid.Vidal	65	9	9	0.134	8.9	1	0.86364
GRSPaV	V.vinifera.var	65	41	41	0.612	40.4	1	0.37879
GRSPaV	V.hybrid.var.	65	4	4	0.06	3.9	1	0.93939
GRSPaV	V.sp.var	65	8	7	0.119	7.9	0.12121	1
GRVFV	GSyV-1	16	12	6	0.044	2.9	0.99443	0.03142
GRVFV	GVE	16	6	0	0.022	1.5	0.17489	1
GRVFV	GVB	16	17	4	0.062	4.1	0.60803	0.64867
GRVFV	GVH	16	9	1	0.033	2.2	0.29976	0.93231
GRVFV	HSVd	16	24	8	0.088	5.8	0.94386	0.15761
GRVFV	Symptom	16	47	11	0.173	11.4	0.51714	0.71962
GRVFV	V.hybrid.Vidal	16	9	1	0.033	2.2	0.29976	0.93231

GRVFV	V.vinifera.var	16	41	13	0.151	9.9	0.98567	0.06171
GRVFV	V.sp.var	16	8	1	0.029	1.9	0.37172	0.90653
GSyV-1	GVE	12	6	0	0.017	1.1	0.28426	1
GSyV-1	GVB	12	17	4	0.047	3.1	0.84819	0.36983
GSyV-1	GVH	12	9	1	0.025	1.6	0.48099	0.85633
GSyV-1	HSVd	12	24	6	0.066	4.4	0.92006	0.22337
GSyV-1	Symptom	12	47	7	0.129	8.5	0.2265	0.92206
GSyV-1	V.hybrid.Vidal	12	9	2	0.025	1.6	0.79678	0.51901
GSyV-1	V.vinifera.var	12	41	6	0.113	7.5	0.26219	0.89957
GSyV-1	V.sp.var	12	8	2	0.022	1.5	0.84795	0.44883
GVE	GVB	6	17	3	0.023	1.5	0.96572	0.17217
GVE	HSVd	6	24	2	0.033	2.2	0.62244	0.71756
GVE	Symptom	6	47	4	0.065	4.3	0.56105	0.77464
GVE	V.vinifera.var	6	41	1	0.056	3.7	0.02592	0.99805
GVB	GVH	17	9	2	0.035	2.3	0.57825	0.73737
GVB	HSVd	17	24	4	0.094	6.2	0.16282	0.94496
GVB	Symptom	17	47	14	0.183	12.1	0.93647	0.19511
GVB	V.hybrid.Vidal	17	9	2	0.035	2.3	0.57825	0.73737
GVB	V.vinifera.var	17	41	9	0.16	10.6	0.26715	0.88346
GVB	V.hybrid.var.	17	4	3	0.016	1	0.9967	0.04953
GVB	V.sp.var	17	8	3	0.031	2.1	0.88965	0.33611
GVH	HSVd	9	24	0	0.05	3.3	0.01205	1
GVH	Symptom	9	47	0	0.097	6.4	0	1
GVH	V.hybrid.Vidal	9	9	6	0.019	1.2	1	7e-05
GVH	V.vinifera.var	9	41	0	0.085	5.6	6e-05	1
GVH	V.sp.var	9	8	0	0.017	1.1	0.2877	1
HSVd	Symptom	24	47	20	0.259	17.1	0.97594	0.08465
HSVd	V.hybrid.Vidal	24	9	0	0.05	3.3	0.01205	1
HSVd	V.vinifera.var	24	41	19	0.226	14.9	0.99343	0.02739
HSVd	V.hybrid.var.	24	4	3	0.022	1.5	0.98526	0.13269

HSVd	V.sp.var	24	8	1	0.044	2.9	0.13328	0.97945
Symptom	V.hybrid.Vidal	47	9	0	0.097	6.4	0	1
Symptom	V.vinifera.var	47	41	38	0.442	29.2	1	0
Symptom	V.hybrid.var.	47	4	3	0.043	2.8	0.75252	0.67495
Symptom	V.sp.var	47	8	5	0.086	5.7	0.41752	0.84127
V.hybrid.Vidal	V.vinifera.var	9	41	0	0.085	5.6	6e-05	1
V.hybrid.Vidal	V.sp.var	9	8	0	0.017	1.1	0.2877	1
V.vinifera.var	V.hybrid.var.	41	4	0	0.038	2.5	0.01755	1
V.vinifera.var	V.sp.var	41	8	0	0.075	5	0.00019	1

Table S2. GenBank accession numbers of grapevine leafroll-associated virus 2 and their nucleotide positions of sequences used for generation of concatenated sequences in this study

Sample*	Isolate	GB acc.	Positions of sequence segments used								
	12G4102	MH814500	1313-2212	3635-4346	5147-5358	6125-6302	6720-6967	10495-10645	12316-13117	13582-14309	14586-16442
	12G4103	MH814498	1298-2212	3635-4346	5147-5358	6125-6302	6720-6967	10492-10644	12315-13116	13581-14308	14582-16436
	ISA-BR	KX774192	1323-2237	3660-4371	5172-5383	6147-6324	6742-6989	10488-10645	12316-13117	13582-14309	14564-16433
	Goldfinger	KU508672	1320-2219	3642-4353	5154-5365	6132-6309	6727-6974	10502-10652	12323-13124	13589-14316	14593-16449
DSJPN1	DSIPN1_GLRaV2-01	MT899925	103-1002	2425-3136	3937-4148	4915-5092	5510-5757	9285-9435	11106-11907	12372-13099	13376-15232
DSJPN2	DSIPN2_GLRaV2-01	MT899926	1278-2177	3600-4311	5112-5323	6090-6267	6685-6932	10460-10610	12281-13082	13547-14274	14551-16407
DSJPN3	DSIPN3_GLRaV2-01	MT899927	1128-2027	3450-4161	4962-5173	5940-6117	6535-6782	10310-10460	12131-12932	13397-14124	14401-16257
DSJPN4	DSIPN4_GLRaV2-01	MT899928	1276-2175	3598-4309	5110-5321	6088-6265	6683-6930	10458-10608	12279-13080	13545-14272	14549-16405

DSJPN5	DSIPN5_GLRaV2-01	MT899929	519-1418	2841-3552	4353-4564	5331-5508	5926-6173	9701-9851	11522-12323	12788-13515	13792-15648
DSJPN9	DSIPN9_GLRaV2-01	MT899930	491-1390	2813-3524	4325-4536	5303-5480	5898-6145	9673-9823	11492-12295	12760-13487	13764-15620

*collected in this study.

Table S3. GenBank accession numbers of grapevine Rupestris stem pitting-associated virus and their nucleotide positions of sequences used for generation of concatenated sequences used in this study

Sample/GB acc.#	Isolate/Strain	Positions of Sequence segments/GB acc.#				
MG938295	28	379-854	1056-1757	2165-3045	3357-6569	7191-8326
AY368590	Syrah	392-867	1069-1771	2179-3058	3370-6582	7204-8339
MG938303	24	394-869	1071-1772	2180-3060	3372-6584	7206-8341
BacPN4*	BacPN4_RSP-01	MT832848	MT832859	MT832870	MT832881	MT855968
BacPN8*	BacPN8_RSP-01	MT832849	MT832860	MT832871	MT832882	MT855969
BacPN9*	BacPN9_RSP-01	MT832850	MT832861	MT832872	MT832883	MT855970
BacSB11*	BacSB11_RSP-01	MT832851	MT832862	MT832873	MT832884	MT855971
BacVd11*	BacVd11_RSP-01	MT832852	MT832863	MT832874	MT832885	MT855972
BIO3_36J*	BIO3_36J_RSP-01	MT832853	MT832864	MT832875	MT832886	MT855973
CO15_56J*	CO15_56J_RSP-01	MT832854	MT832865	MT832876	MT832887	MT855974
DM_85*	DM_85_RSP-01	MT832855	MT832866	MT832877	MT832888	MT855975
BacMF3*	BacMF3_RSP-01	MT832856	MT832867	MT832878	MT832889	MT855976
BacMF6*	BacMF6_RSP-01	MT832857	MT832868	MT832879	MT832890	MT855977
FrTM6_75J*	FrTM6_75J_RSP-01	MT832858	MT832869	MT832880	MT832891	MT855978

*collected in this study.
